# Prevalence and characteristics of dry eye disease in Parkinson’s disease: a systematic review and meta-analysis

**DOI:** 10.1038/s41598-022-22037-y

**Published:** 2022-11-01

**Authors:** Ken Nagino, Jaemyoung Sung, Genko Oyama, Motoshi Hayano, Nobutaka Hattori, Yuichi Okumura, Kenta Fujio, Yasutsugu Akasaki, Tianxiang Huang, Akie Midorikawa-Inomata, Keiichi Fujimoto, Atsuko Eguchi, Shokirova Hurramhon, Maria Miura, Mizu Ohno, Kunihiko Hirosawa, Yuki Morooka, Akira Murakami, Hiroyuki Kobayashi, Takenori Inomata

**Affiliations:** 1 Department of Hospital Administration, Juntendo University Graduate School of Medicine, Tokyo, Japan; 2 Department of Digital Medicine, Juntendo University Graduate School of Medicine, Tokyo, Japan; 3 Department of Ophthalmology, Juntendo University Graduate School of Medicine, 2-1-1 Hongo, Bunkyo-ku, Tokyo, 113-0033 Japan; 4 Department of Neurology, Juntendo University Graduate School of Medicine, Tokyo, Japan; 5 Department of Neuropsychiatry, Keio University School of Medicine, Tokyo, Japan; 6 Juntendo University Graduate School of Medicine, AI Incubation Farm, Tokyo, Japan

**Keywords:** Eye manifestations, Corneal diseases, Parkinson's disease

## Abstract

We investigated and characterized the prevalence of dry eye disease (DED) in Parkinson’s disease (PD). PubMed and EMBASE databases were searched for relevant studies between January 1, 1979 and March 10, 2022. Quality was assessed using the Joanna Briggs Institute Critical Appraisal Checklist. Study-specific estimates were combined using the DerSimonian–Laird random-effects model. Prevalence of subjective DED symptoms in patients with PD and mean differences in blink rate, corneal thickness, tear film breakup time, and tear secretion volume on Schirmer test I were compared to those in controls. Of 383 studies, 13 (1519 patients with PD) and 12 were included in qualitative and quantitative syntheses, respectively. Meta-analysis revealed a 61.1% prevalence of subjective DED symptoms in PD and that, compared with controls, patients with PD had significantly lower blink rate, thinner corneal thickness, shorter tear film breakup time, and lower tear secretion volumes on Schirmer test I, without and with anesthesia.

## Introduction

Parkinson’s disease (PD) is the second most common neurodegenerative disease, following Alzheimer’s disease^1,2^. The prevalence of PD is estimated to be 0.3% in the entire population and 1% in individuals over 60 years old, with a projected increase in the population^1–3^. PD is characterized by dopamine deficiency in the striatum due to the degeneration of dopaminergic neurons in the substantia nigra and accumulation of alpha-synuclein^4,5^. Patients with PD exhibit typical motor symptoms, such as bradykinesia, rigidity, and tremors, as well as various non-motor symptoms^6,7^, including depression, sleep disorders, dementia, constipation, olfactory disturbances, autonomic neuropathy, and ocular dysfunction^2–4,6–8^.

Dry eye disease (DED) is a common ocular disease^9^ with a global prevalence of 5–50%, which is expected to increase with the aging society^9–11^. The symptoms of DED range from eye discomfort to chronic vision loss, resulting in decreased quality of life and work productivity, thus imposing an economic burden on society^10,12–14^. The key pathophysiology of DED is the disruption of tear film homeostasis due to several factors, including eyelid and blink abnormalities or tear component deficiency^9,15,16^. Blinking distributes mucin lipids and tears to the ocular surface to maintain moistness and protect the eye from external stimuli^17–20^. Dopamine deficiency in PD patients leads to decreased blink rate (BR) and increased abnormal blinking^21,22^. However, a recent study has reported that BR is not associated with dopamine synthesis capacity^23^ Decreased BR and increased incomplete blinking in patients with PD may cause tear film hyperosmolality, accelerated tear evaporation, and corneal damage^15,24,25^. Tear secretion is regulated primarily by the parasympathetic nervous system^13^, and the tear secretory function is often suppressed by autonomic dysfunction in PD due to PD-associated neurodegeneration^13,26,27^.

The prevalence of DED in patients with PD is estimated to be 53.0–60.0% worldwide^26,28,29^. Patients with PD are at high risk of DED comorbidity due to neurodegeneration, which causes abnormal eyelid motility, decreased BR, and tear deficiency^29–31^. However, patients with PD with reduced corneal sensitivity have decreased subjective DED symptoms and often do not present with complaints suggestive of DED^32^. Moreover, there is a lack of established interdisciplinary systems in neurology and ophthalmology, which include DED examinations in patients with PD. Further, there is a paucity of DED examination data on patients with PD, which has obscured the accurate evaluation of DED in these patients.

Therefore, this study aimed to estimate the prevalence and characterize the clinical findings of DED in patients with PD and provide evidence on the pathophysiology of DED, emphasizing its comorbidity with PD.

## Results

### Search results

**Figure 1:**
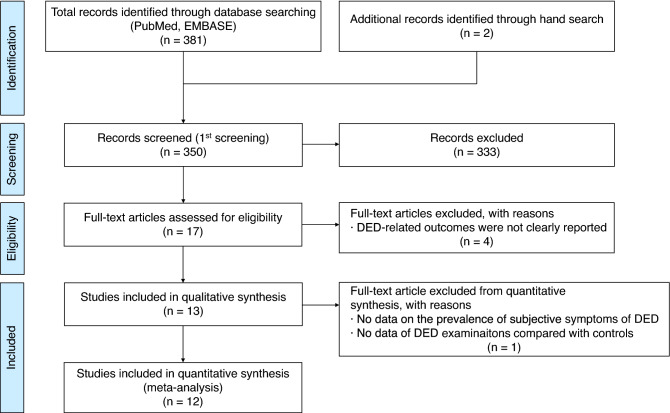
Flow diagram of study selection. *DED* dry eye disease.

The database search identified 381 articles and two additional articles obtained after a manual search^33^. After removing 33 duplicates, the title, abstract, and article type of the remaining 350 articles were reviewed. After the initial screening, 333 articles were further excluded since they were irrelevant to the topic (n = 85) or ineligible article types (n = 248). The full text of the 17 remaining articles was reviewed. Four articles did not clearly report DED-related outcomes and were excluded; hence, 13 articles were included in the qualitative synthesis. Furthermore, one article did not report on the prevalence of subjective DED symptoms or compare DED examination results of patients with PD with those of a control group; thus, 12 articles were included in the quantitative synthesis of the meta-analysis, as shown in Fig. 1.

### Study and demographic characteristics

The 13 studies (1519 patients with PD and 639 controls) included in the systematic review were published from January 27, 2004, to March 9, 2022^26,29–40^. Four studies were published in the United Kingdom^30,31,36,39^, three in Turkey^35,37,38^, two in the United States^29,32^, two in two combined countries (Netherlands–Austria)^33,34^, and one each in Poland^26^ and Thailand^40^. Three studies reported only subjective DED symptoms or compared DED examination results of patients with PD with those of a control group^33,36,40^. The mean age of patients in 12 articles ranged from 60.6 to 70.0 years^26,30,32,34–40^, and one article reported a median age of 68.0 years^33^. The mean duration of PD in 10 articles ranged from 1.8 to 11.4 years^26,29,30,33,35–40^, and one article reported a median of 6.0 years^33^. Eleven studies reported the number of men and women, totaling 962 men and 747 women^26,29,30,33,35–40^. All the 13 articles were cross-sectional studies, with 12 articles rated as high quality and one as moderate quality (Table 1)^31^.

**Table 1:** Characteristics of the included studies.

Source	Publication date	Study type	Country	Participants’ characteristics			Patients with PD			Reported DED-related outcomes	Quality score (0–8)^†^
				Population health status	Sample size, n (M/F)	Age (year)*	HYS (1–5)*	PD duration, year*	Patients with DED subjective symptoms, n (%)		
Borm et al.^33^	March 9, 2022	Prospective cross-sectional study	The Netherlands & Austria	Patients with PD	69/33	Median, 68.0	Median, 2.0	Median, 6.0	NA	Blink rate, TFBUT, Schirmer test I with anesthesia, corneal thickness	8 (high)
Ulusoy et al.^35^	May 4, 2020	Prospective cross-sectional study	Turkey	Patients with PD & healthy controls	PD, 34/30, controls, 44/20	PD, 65.2, controls, 63.2	2.8	5.6	NA	Blink rate, TFBUT, Schirmer test I with anesthesia, corneal power, corneal thickness, corneal volume, axial length, endothelial cell density	7 (high)
Borm et al.^34^	April 7, 2020	Prospective cross-sectional study	The Netherlands & Austria	Patients with PD & healthy controls	PD, 467/381, controls, 125/125	Median, PD, 70.0, controls, 70.0	NA	7.0	534 (67%)	DED subjective symptoms	7 (high)
Leclair-Visonneau et al.^36^	April 1, 2019	Prospective cross-sectional study	UK	Patients with PD	28/15	60.6	NA	8.9	19 (44.2%)	DED subjective symptoms	7 (high)
Samart^40^	December 1, 2018	Prospective cross-sectional study	Thailand	Patients with PD	54/56	65.7	2.1	7	64 (58.2)	DED subjective symptoms	6 (high)
Demirci S et al.^37^	April 7, 2016	Prospective cross-sectional study	Turkey	Patients with PD & healthy controls	PD, 26/14, controls, 27/13	PD, 64.9, controls, 62.0	2.1	6.2	NA	Blink rate, TFBUT, Schirmer test I with anesthesia, CFS, OSDI, corneal power, corneal thickness, corneal volume	7 (high)
Sogutlu Sari et al.^30^	August 5, 2015	Prospective cross-sectional study	UK	Patients with PD & healthy controls	PD, 20/17, controls, 14/23	PD, 67.4, controls, 65.1	1.7	5.7	NA	Blink rate, TFBUT, Schirmer test I without anesthesia, tear osmolarity	8 (high)
Nowacka et al.^26^	November 11, 2014	Prospective cross-sectional study	Poland	Patients with PD & healthy controls	PD, 56/44, controls, 56/44	PD, 68.5, controls, 68.6	1.8	5.6	NA	TFBUT, Schirmer test I without anesthesia, OSDI, tear osmolarity, meibomian gland dysfunction, lid-parallel conjunctival folds	8 (high)
Ornek et al.^38^	June 23, 2014	Prospective cross-sectional study	Turkey	Patients with PD & healthy controls	PD, 12/18, controls, 15/23	PD, 65.1, controls, 65.3	NA	11.4	NA	TFBUT, Schirmer test I without anesthesia, corneal sensitivity	7 (high)
Aksoy et al.^39^	April 1, 2014	Prospective cross-sectional study	UK	Patients with PD & healthy controls	PD, 32/23, controls, 23/17	PD, 63.4, controls, 61.1	2.2	4.8	NA	Blink rate, TFBUT, Schirmer test I with anesthesia, corneal thickness	8 (high)
Reddy et al.^32^	May, 2013	Prospective cross-sectional study	USA	Patients with PD & healthy controls	PD, 4, controls, 5 (No sex data)	PD, 65.0, controls, 57.0	3.0	NA	1 (25%)	DED subjective symptoms, blink rate TFBUT, corneal sensitivity, endothelial cell density	6 (high)
Tamer et al.^31^	April 25, 2005	Prospective cross-sectional study	UK	Patients with PD & healthy controls	PD, 56, controls, 34 (No sex data)	PD, 64.1, controls, 62.4	NA	NA	49 (87.5)	DED subjective symptoms, blink rate TFBUT, Schirmer test I without anesthesia, CFS, corneal-Rose Bengal staining	4 (moderate)
Biousse et al.^29^	January 27, 2004	Prospective cross-sectional study	USA	Patients with PD & healthy controls	PD, 23/7, controls, 11/20	PD, 61.0, controls, 58.0	NA	1.8	NA	Blink rate, TFBUT, Schirmer test I with anesthesia, corneal-Rose Bengal staining	5 (high)

*PD* Parkinson’s disease, *DED* dry eye disease, *HYS* Hoehn and Yahr Scale, *TFBUT* tear film breakup time, *CFS* corneal fluorescein staining, *OSDI* Ocular Surface Disease Index, *NA* not applicable.

*Age, HYS, and PD duration represent means unless otherwise noted.

^†^Quality score was assessed using the Joanna Briggs Institute critical appraisal checklist for Analytical Cross-Sectional Studies.

### Prevalence of subjective DED symptoms in patients with PD

**Figure 2:**
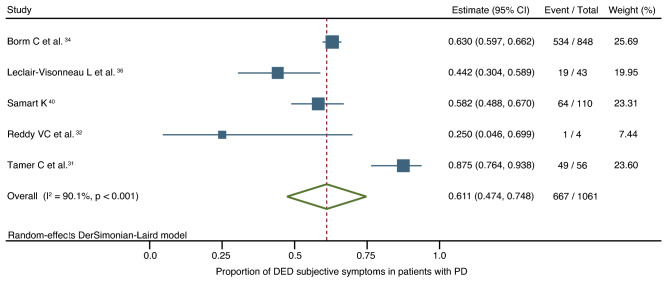
Forest plot of the prevalence of subjective DED symptoms in patients with PD. *DED* dry eye disease, *PD* Parkinson’s disease, *CI* confidence interval.

Five studies reported the prevalence of subjective DED symptoms in a total of 1,061 patients with PD^31,32,34,36,40^. Among these patients, 61.1% of them (667/1061 cases; confidence interval [CI] 47.4–74.8) reported subjective DED symptoms. This outcome reflected high and significant heterogeneity (I^2^ = 91.1%, *P* < 0.001). Subgroup analysis was not performed as only one study had a group with a Hoehn and Yahr Scale (HYS) score ≥ 2.5 (Fig. 2)^32^.

### DED examination of patients with PD versus controls

#### Blink rate

**Figure 3:**
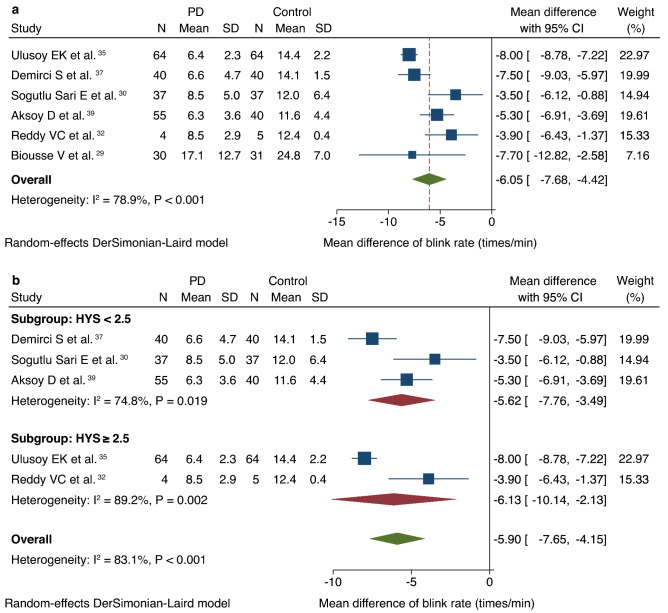
Forest plot of the blink rate of patients with PD versus controls. (**a**) Forest plot of the blink rate. (**b**) Forest plot of the blink rate according to subgroups based on HYS scores. *PD* Parkinson’s disease, *SD* standard deviation, *CI* confidence interval, *HYS* Hoehn and Yahr Scale.

Six studies compared BR between patients with PD (n = 230) and controls (n = 217)^29,30,32,35,37,39^. BR was significantly lower in patients with PD than in controls (mean difference [MD], − 6.1 times/min; CI − 7.7 to − 4.4; Fig. 3a). As significant heterogeneity (I^2^ = 78.9%, *P* < 0.001) was observed in the analysis, a subgroup analysis based on the HYS scores was conducted, which excluded one study that did not report HYS scores^29^. Subgroup analysis showed that BR of patients with PD in the HYS score < 2.5 and HYS score ≥ 2.5 subgroups were significantly lower than that of the controls (HYS score < 2.5, MD, − 5.6 times/min; CI − 7.8 to − 3.5 and HYS score ≥ 2.5, MD, − 6.1 times/min; CI − 10.1 to − 2.1, respectively; Fig. 3b). The analysis revealed that the heterogeneity in the HYS score < 2.5 subgroup remained mostly high, while that in the HYS score ≥ 2.5 subgroup remained high (HYS score < 2.5, I^2^ = 74.8%, *P* = 0.019 and HYS score ≥ 2.5, I^2^ = 89.2%, *P* = 0.002, respectively; Fig. 3b). Publication bias was not observed (Begg’s test: *P* = 0.133; Egger’s test: *P* = 0.287).

#### Corneal thickness

**Figure 4:**
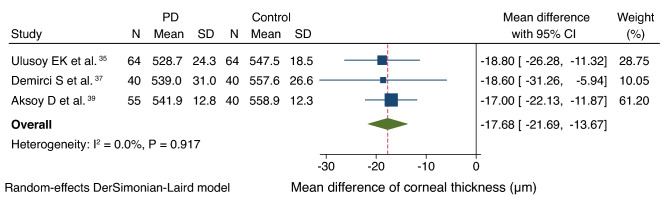
Forest plot of the corneal thickness of patients with PD versus controls. *PD* Parkinson’s disease, *SD* standard deviation, *CI* confidence interval.

Three studies reported on corneal thickness^35,37,39^. Compared with the controls (144 patients), patients with PD (199 patients) had significantly thinner corneal thickness (MD, − 17.7 µm; CI − 21.7 to − 13.7). There was no heterogeneity among the studies (I^2^ = 0.0%, *P* = 0.917; Fig. 4). Publication bias was not observed (Begg’s test: *P* = 1.000; Egger’s test: *P* = 0.756).

#### Tear film breakup time

**Figure 5:**
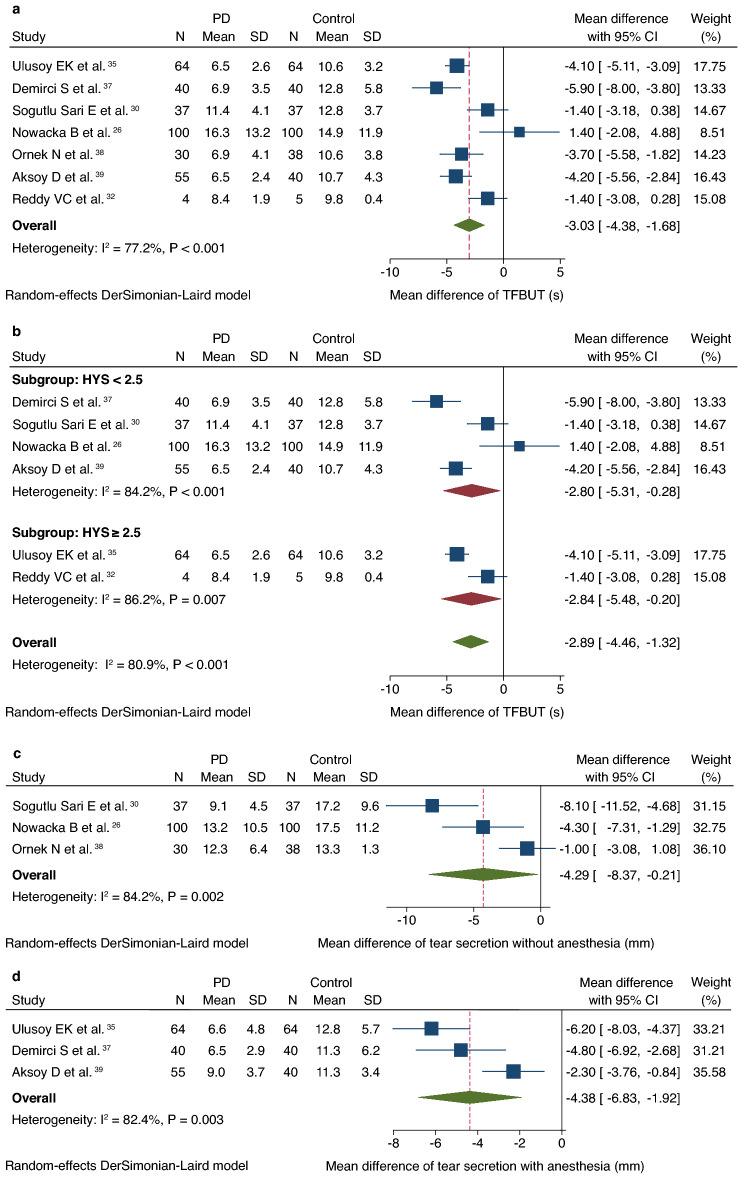
Forest plot of DED clinical examinations in patients with PD versus controls. (**a**) Forest plot of TFBUT. (**b**) Forest plot of TFBUT according to subgroups based on HYS scores. (**c**) Forest plot of scores of Schirmer test I without anesthesia. (**d**) Forest plot of Schirmer test I scores with anesthesia. *PD* Parkinson’s disease, *SD* standard deviation, *CI* confidence interval, *HYS* Hoehn and Yahr Scale, *TFBUT* tear film breakup time.

The tear film breakup time (TFBUT) in seven studies, comprising a total of 330 patients with PD and 324 controls, was evaluated^26,30,32,35,37–39^. TFBUT was significantly lower in patients with PD than in controls (MD, − 3.0 s; CI − 4.4 to − 1.7; Fig. 5a). Due to the significant heterogeneity (I^2^ = 77.2%, *P* < 0.001) observed, a subgroup analysis was performed, which excluded one study that did not report HYS scores^38^. Subgroup analysis showed that TFBUT of patients with PD in the HYS score < 2.5 and HYS score ≥ 2.5 subgroups were significantly lower than that of the controls (HYS score < 2.5, MD, − 2.8 s; CI − 5.3 to − 0.3 and HYS score ≥ 2.5, MD − 2.8 s; CI − 4.5 to − 1.3, respectively; Fig. 5b). Heterogeneity in the HYS score < 2.5 and ≥ 2.5 subgroups remained high (HYS score < 2.5, I^2^ = 84.2%, *P* = 0.001 and HYS score ≥ 2.5, I^2^ = 86.2%, *P* = 0.007, respectively; Fig. 5b). Publication bias was not observed (Begg’s test: *P* = 0.548; Egger’s test: *P* = 0.092).

#### Schirmer test I

Six studies reported Schirmer test I results in patients with PD and controls. Among these studies, three performed the test without anesthesia (PD, n = 167 and controls, n = 175; Fig. 5c)^26,30,38^, while the remaining three used anesthesia (PD, n = 159 and controls, n = 144; Fig. 5d)^35,37,39^. Tear secretion volume was significantly decreased in patients with PD who did (MD, − 4.3 mm; CI − 8.4 to − 0.2) or did not (MD, − 4.4 mm; CI − 6.8 to − 1.9) receive anesthesia. Despite the significant heterogeneity, regardless of the method used (without anesthesia, I^2^ = 84.2%, *P* = 0.002; with anesthesia, I^2^ = 82.4%, *P* = 0.003), subgroup analysis was not performed due to the small number of studies in each subgroup. Publication bias was not identified (without anesthesia, Begg’s test: *P* = 0.296 and Egger’s test: *P* < 0.001; with anesthesia, Begg’s test: *P* = 1.000 and Egger’s test: *P* = 0.328).

## Discussion

Due to the challenges in coordinating multidisciplinary partnerships to establish robust cross-sectional data on DED in patients with PD, there is a paucity of data on the potential association between DED and PD. Thus, we performed the first systematic review and meta-analysis investigating the prevalence of subjective symptoms and clinical findings of DED in patients with PD. Our results indicated that 61.1% of patients with PD exhibited subjective DED symptoms. Additionally, patients with PD exhibited significantly decreased BR, corneal thickness, TFBUT, and tear secretion compared with those exhibited by healthy controls. These results highlight the possibility of the comorbid occurrence of DED with PD, emphasizing the need for early screening and appropriate interventions by clinicians treating patients with PD.

This meta-analysis revealed that patients with PD, compared to healthy controls, may more likely present with DED^9,41,42^. Various hypotheses have been proposed to explain the association between PD and DED, such as corneal hypoesthesia causing decreased BR and reflex lacrimation, autonomic neuropathy due to anti-dopaminergic dysregulation leading to decreased tear secretion, increased tear osmolarity, decreased tear mucin, and lipid layer disruption secondary to meibomian gland dysfunction^22,26,29–32,43–46^. Impairments in daily activities due to ocular diseases, including DED, cataract, glaucoma, retinal degeneration, diplopia, visual hallucinations, and amblyopia, were reported by 44% of patients with PD^33^. Moreover, DED appears to be one of the ocular causes of decreased quality of life among patients with PD^33^. Nevertheless, due to insufficient reports on the comorbid occurrence of DED in patients with PD, initiatives to establish a multidisciplinary care system, wherein regular ocular examinations and DED screening are incorporated within the routine management of PD, are limited. Delayed DED diagnosis and intervention can eventually cause corneal perforation and scarring, leading to severe chronic ocular and visual impairments^33,47^. Our finding of increased DED prevalence within this population highlights the need for healthcare providers to be vigilant regarding comorbid DED and regularly offer DED screening and intervention for patients with PD.

As PD progresses, alpha-synuclein accumulation spreads from the dopaminergic system in the midbrain to the cortex, resulting in changes that manifest as cognitive and sensory symptoms in addition to motor symptoms^5,48^. BR has been reported to correlate positively with dopaminergic neuron activation^22^. Hence, decreased dopaminergic activity in patients with PD may lead to a decrease in BR, which is consistent with previous reports that suggest an association between decreased BR and corneal hypoesthesia in patients with PD^32,49^. However, a recent study has reported no positive relationship between BR and dopamine synthesis capacity^23^. Nevertheless, our meta-analysis (Fig. 3a) supported the association between BR and PD, indicating that BR was significantly reduced in patients with PD. In addition, the subgroup analysis based on HYS scores showed the significant reduction of BR in each subgroup. As BR and HYS scores are both affected by dopaminergic medications and circadian changes, further comprehensive longitudinal research is warranted to elucidate the relationship between PD and DED-related physiologic changes^44,50^.

Corneal thinning in DED is driven by immunologic activities, chronic dryness, and increased tear osmolarity^37,51,52^. Evidence suggests that the severity of PD has a weak clinical correlation with changes in corneal thickness^37^. Although corneal thinning was observed in our analysis, the mean corneal thicknesses for both patients with PD and controls were within the normal range (patients with PD: 528.7–541.9 µm; controls: 547.5–558.9 µm). Further investigations should pursue the implications of the degree of corneal thinning on ocular function in patients with PD^53^. Nonetheless, our findings emphasize the need for clinicians to be vigilant regarding falsely suppressed intraocular pressure measurements caused by thinner corneas in patients with PD. Clinicians should adopt appropriate management approaches for disease processes associated with thinner corneas, such as open-angle glaucoma^54,55^.

From the observed decrease in TFBUT, we can imply that decreased BR, incomplete blinks, and tear film instability are caused by the disruption of the aqueous, lipid, and mucin composition in patients with PD. These events may originate from nervous system dysregulation associated with PD progression^31,45,56^. Additionally, patients with PD exhibit a more severe meibomian gland dysfunction, which is critical for maintaining tear film stability and homeostasis^26^. Our results align with previous findings, with a notable decrease in TFBUT in the PD cohort than in the controls. The subgroup analysis based on HYS scores in this study showed significant reduction of TFBUT in the HYS score < 2.5 and ≥ 2.5 subgroups (HYS score < 2.5, MD, − 2.8 s; CI − 5.3 to − 0.3 and HYS score ≥ 2.5, MD, − 2.8 s; CI − 4.5 to − 1.3, respectively). This result indicated that PD was associated with decreased TFBUT. Decreased TFBUT has been identified as a key marker of visual impairment^56^, which has strong implications for overall cognitive and motor function in patients with PD^57^.

Tear secretion is mainly regulated by the autonomic nervous system^35^. Autonomic dysregulation observed in PD causes disturbances in proper tear production and secretion^35^. Decreased corneal sensitivity in PD leads to a decrease in reflex lacrimation^30,37^ and is further exacerbated by the frequent administration of medications with anticholinergic properties^58^, such as trihexyphenidyl and amantadine. In addition, diabetes and Sjögren's syndrome, which are considered risk factors for PD, also trigger decreased tear secretion^59,60^. Our meta-analysis revealed a significant, near-equivalent decrease in Schirmer test scores with or without anesthesia in the PD cohort, supporting the hypothesis that PD induces decreases in both baseline and reflex lacrimation^16^. Moreover, the aqueous-deficient subtype of DED is associated with increased severity due to elevated friction between the corneal surface and palpebral conjunctiva^61^. Therefore, early intervention may be necessary to prevent visual impairment, as well as resultant cognitive and motor function decline.

This study has several limitations. The prevalence of DED was based on subjective symptoms reported by patients, which may have discrepancies with the other objective clinical markers of DED diagnosis^62^. Additionally, our meta-analysis identified significant heterogeneity in the included studies; thus, our results should be interpreted cautiously. Furthermore, adjustments for heterogeneity were not made based on the HYS scores in the subgroup analysis. Possible explanations for such heterogeneity include variability in patient demographics, sample size, and pharmacological influences. We also noted the possibility of sampling bias due to the limited geographical coverage of the included studies, given that all included studies were performed in North America or Europe, except for one publication from Thailand. Finally, all included studies had a cross-sectional design, which limits the establishment of temporal or cause-and-effect relationships in the identified differences in DED parameters between the controls and patients with PD. Our study identified the characteristics of DED in patients with PD. Previous studies on DED in PD patients mainly focused on a small number of cases. Future large-scale studies on DED in patients with PD are expected to elucidate the pathogenesis of DED in patients with PD. In addition, the characteristics of DED subtypes in patients with PD and the effect of PD treatment on DED in patients with PD have not been elucidated.

In conclusion, our study revealed that 61.1% of patients with PD exhibited subjective DED symptoms. Our findings emphasize the need for clinicians to be vigilant of the presence of DED when managing patients with PD and to appropriately adjust the threshold for monitoring and early intervention in these high-risk populations.

## Methods

### Search strategy

This study was performed in accordance with the Preferred Reporting Items for Systematic Reviews and Meta-Analyses guidelines^63^. PubMed and EMBASE databases were searched on March 10, 2022, for articles published from January 1, 1979, to March 10, 2022. A literature search was conducted using terms in free text and medical subject headings (MeSH in PubMed and Emtree in EMBASE) without restriction. The specific search terms were [PubMed: ((Parkinson Disease [MeSH Terms]) OR (Parkinson)) AND ((dry eye syndromes [MeSH Terms]) OR (dry eye))], [EMBASE: (“Parkinson disease”/exp OR “Parkinson”) AND (“dry eye syndrome”/exp OR “dry eye”)]. The inclusion and exclusion criteria are presented in Table 2.

**Table 2:** Study inclusion and exclusion criteria.

**Inclusion criteria**	
1	Population: patients with PD evaluated for DED
2	Study design: retrospective (cross-sectional studies, case–control studies, case series, and case reports) and prospective studies
3	Outcome: number, age, sex, PD duration, Hoehn and Yahr Scale, blink rate, corneal thickness, tear film breakup time, Schirmer test I, and prevalence of DED subjective symptoms
**Exclusion criteria**	
1	Ineligible article types: editorials, letters, commentaries, book chapters, reviews, systematic reviews, conference proceedings, and conference abstracts
2	Animal-based studies
3	Studies on non-PD controls presenting with parkinsonism
4	DED-related outcomes were not clearly reported
5	Unrelated topics

*PD* Parkinson’s disease, *DED* dry eye disease.

### Data extraction

Search results were compiled using EndNote 20.2 software (Clarivate Analytics, Philadelphia, PA, USA). An initial screening of titles, abstracts, and full texts of retrieved articles was performed by two independent reviewers (K.N. and T.I.). Subsequently, the eligible articles were determined by consensus. A standardized data extraction sheet was used to extract information, and the results were cross-checked. Inter-reviewer disagreements regarding the extracted data were resolved through discussions with another reviewer (J.S.). The following data were extracted: first author’s name, date of publication, type of study (retrospective or prospective), country, population health status, sample size, age, sex, HYS score, PD duration, DED examinations (BR, corneal thickness, TFBUT, Schirmer test I, and prevalence of subjective symptoms of DED).

### Study quality assessment

The quality of the selected studies was assessed using the Joanna Briggs Institute Critical Appraisal Checklist for Analytical Cross-Sectional Studies^64^. The questionnaire consisted of eight questions answered with the following responses: “yes,” “no,” “unknown,” or “not applicable.” Literature quality was determined based on the total number of “yes” responses, with ≥ 5, 3–4, and 0–2 indicating high, moderate, and low quality, respectively.

### PD and DED evaluation outcomes

#### Hoehn and Yahr scale

The HYS is an index of movement disorder severity in patients with PD and includes stages 1–5, with stage 5 being the most severe^65,66^. Each stage is described as follows: stage 1, only unilateral involvement, typically with minimal or no functional disability; stage 2, bilateral or midline involvement without impairment of balance; stage 3, bilateral disease and mild to moderate disability with impaired postural reflexes; stage 4, severely disabling disease but still able to walk or stand unassisted; and stage 5, confinement to the bed or wheelchair unless aided^66^.

### DED examinations

The prevalence of subjective DED symptoms was assessed using disease-specific questionnaires, including the Visual Impairment in Parkinson’s Disease Questionnaire^34^, Thammasat University Non-Motor Symptoms Questionnaire^40^, a two-choice question (yes/no) regarding subjective DED symptoms^32,36^, and a question regarding the frequency of those symptoms (the response was positive if the symptoms occurred at least once a month)^31^. Patients who responded positively to the dichotomous question regarding DED symptoms were considered to have DED symptoms. The results of the DED examinations, including the number of blinks per minute^35^, central corneal thickness measured using an ultrasonic pachymeter or the Pentacam Scheimpflug system, TFBUT^67^, and tear secretion volume determined using the Schirmer test I^68^ (performed with or without topical anesthesia), were extracted^69^.

### Statistical analyses

MDs with 95% CIs were used in the meta-analysis of continuous outcomes of two-arm studies. The prevalence of subjective DED symptoms in patients with PD with CI was combined using one-group meta-analysis. All analyses were conducted using the DerSimonian–Laird random-effects model. The study heterogeneity was assessed using the I^2^ statistic and the chi-square test. I^2^ > 75% was considered high heterogeneity, and *P* < 0.05 indicated significant heterogeneity. Subgroup analysis was conducted based on HYS for high and significant heterogeneity outcomes. Each subgroup was defined as HYS < 2.5 and HYS ≥ 2.5. Subgroup analyses were performed when each subgroup included two or more studies. Egger’s and Begg’s tests were performed to estimate the publication bias of continuous outcomes, with *P* < 0.05 in both tests indicating significant publication bias. The STATA software package (v 16.1, Stata Corp, College Station, TX, USA) with the *metaprop* command was used for all analyses^70^.

## Data Availability

All data are available in the main text or the supplementary materials. Data access, responsibility, and analysis: Takenori Inomata, had full access to all the data in the study and take responsibility for the integrity of the data and the accuracy of data analysis.
